# Supplementary material for: OpenMM 7: Rapid development of high performance algorithms for molecular dynamics
Source: PLoS Comput Biol. 2017 Jul 26;13(7):e1005659. doi: 10.1371/journal.pcbi.1005659 (PMC5549999; doi:10.1371/journal.pcbi.1005659)
Supplement: S1 Source Code — (ZIP) [file pcbi.1005659.s002.zip › OpenMM-7.0.1-Source/docs/api-python/generated/simtk.openmm.openmm.TwoParticleAverageSite.html]

TwoParticleAverageSite — OpenMM 7.0.1.dev-5e86c4f documentation


# TwoParticleAverageSite¶

*class* `simtk.openmm.openmm.``TwoParticleAverageSite`(*\*args*)¶
:   This is a VirtualSite that computes the particle location as a weighted average of two other particle’s locations. Assuming the weights add up to 1, this means the virtual site is on the line passing through the two particles.

    `__init__`(*self*, *particle1*, *particle2*, *weight1*, *weight2*) → TwoParticleAverageSite¶
    :   \_\_init\_\_(self, other) -> TwoParticleAverageSite

        Create a new TwoParticleAverageSite virtual site. Normally weight1 and weight2 should add up to 1, although this is not strictly required.

        |  |  |
        | --- | --- |
        | Parameters: | - **particle1** (*int*) – the index of the first particle - **particle2** (*int*) – the index of the second particle - **weight1** (*double*) – the weight factor (between 0 and 1) for the first particle - **weight2** (*double*) – the weight factor (between 0 and 1) for the second particle |

    Methods

    |  |  |
    | --- | --- |
    | `__init__`((self, particle1, particle2, ...) | \_\_init\_\_(self, other) -> TwoParticleAverageSite |
    | `getNumParticles`((self) -> int) | Get the number of particles this virtual site depends on. |
    | `getParticle`((self, particle) -> int) | Get the index of a particle this virtual site depends on. |
    | `getWeight`((self, particle) -> double) | Get the weight factor used for a particle this virtual site depends on. |

    `getWeight`(*self*, *particle*) → double¶
    :   Get the weight factor used for a particle this virtual site depends on.

        |  |  |
        | --- | --- |
        | Parameters: | **particle** (*int*) – the particle to get (between 0 and getNumParticles()) |
        | Returns: | the weight factor used for that particle |
        | Return type: | double |

    `__delattr__`¶
    :   x.\_\_delattr\_\_(‘name’) <==> del x.name

    `__format__`()¶
    :   default object formatter

    `__getattribute__`¶
    :   x.\_\_getattribute\_\_(‘name’) <==> x.name

    `__hash__`¶

    `__reduce__`()¶
    :   helper for pickle

    `__reduce_ex__`()¶
    :   helper for pickle

    `__sizeof__`() → int¶
    :   size of object in memory, in bytes

    `__str__`¶

    `getNumParticles`(*self*) → int¶
    :   Get the number of particles this virtual site depends on.

    `getParticle`(*self*, *particle*) → int¶
    :   Get the index of a particle this virtual site depends on.

        |  |  |
        | --- | --- |
        | Parameters: | **particle** (*int*) – the particle to get (between 0 and getNumParticles()) |
        | Returns: | the index of the particle in the System |
        | Return type: | int |

High performance molecular simulation on GPUs


### Navigation

- Application Layer
- Library Layer
  - Core Objects
  - Forces
  - Integrators
  - Extras

©2015, Stanford University and the Authors.
|
Powered by Sphinx 1.3.1
& Alabaster 0.7.7
|
Page source
